# Natural History of Histopathologic Changes in Cardiomyopathy of Golden Retriever Muscular Dystrophy

**DOI:** 10.3389/fvets.2021.759585

**Published:** 2022-02-17

**Authors:** Sarah M. Schneider, Garett T. Sansom, Lee-Jae Guo, Shinji Furuya, Brad R. Weeks, Joe N. Kornegay

**Affiliations:** ^1^Department of Veterinary Pathobiology, Texas A&M University, College Station, TX, United States; ^2^Department of Environmental and Occupational Health, Texas A&M University, College Station, TX, United States; ^3^Department of Veterinary Integrative Biosciences, Texas A&M University, College Station, TX, United States

**Keywords:** cardiomyopathy, dystrophinopathy, golden retriever muscular dystrophy (GRMD), duchenne muscular dystrophy (DMD), natural history

## Abstract

**Background:**

Duchenne muscular dystrophy (DMD) is an X-linked inherited myopathy that causes progressive skeletal and cardiac muscle disease. Heart lesions were described in the earliest DMD reports, and cardiomyopathy is now the leading cause of death. However, diagnostics and treatment for cardiomyopathy have lagged behind those for appendicular and respiratory skeletal muscle disease. Most animal model studies have been done in the mdx mouse, which has a relatively mild form of cardiomyopathy. Dogs with the genetically homologous condition, Golden Retriever muscular dystrophy (GRMD), develop progressive cardiomyopathy analogous to that seen in DMD. Previous descriptive studies of GRMD cardiomyopathy have mostly been limited to selective sampling of the hearts from young dogs.

**Methods and Results:**

We systematically assessed cardiac lesions in 31 GRMD and carrier dogs aged 3 to 76 months and a separate cohort of 2–10-year-old normal hounds. Both semi-quantitative lesion scoring and quantitation of the cross-sectional area of fibrosis distinguished dogs with GRMD disease from normal dogs. The carriers generally had intermediate involvement but had even greater fibrosis than GRMD dogs. Fatty infiltration was the most prominent feature in some older GRMD dogs. Vascular hypertrophy was increased in GRMD dogs and correlated positively with lesion severity. Purkinje fiber vacuolation was also increased but did not correlate with lesion severity. Histopathologic changes correlated with late gadolinium enhancement on cardiac MRI.

**Conclusion:**

These features are generally compatible with those of DMD and further validate GRMD as a useful model to study cardiomyopathy pathogenesis and treatment. Additionally, the nature of some degenerative lesions suggests that functional hypoxia or non-thrombotic ischemia may contribute to disease progression.

## Introduction

Duchenne muscular dystrophy (DMD) and Golden Retriever muscular dystrophy (GRMD) are genetically homologous, phenotypically analogous degenerative muscle diseases caused by mutations in the *DMD* gene, which encodes the dystrophin protein (1, 2). Loss of dystrophin at the sarcolemma leads to membrane fragility and both skeletal (3) and cardiac muscle lesions (4, 5). Cardiac complications are the leading cause of death in young men with DMD (6).

Although the heart and skeletal muscle both lack dystrophin, differences in Ca^2+^ homeostasis (7–9), metabolism (10), and dystrophin–glycoprotein complex localization (11–13) contribute to variable pathologic changes in the two tissues. While some studies have shown that skeletal and cardiac muscle functional deficits track together, others have demonstrated variable progression (5, 6, 14–16). This potential for discordance has clinical significance. Features typical of cardiac disease may not be present in wheelchair-bound DMD boys (17, 18), precluding the assessment of treatment effects on the heart. More importantly, improvement in skeletal muscle function alone could increase strain on the heart and accelerate cardiac disease progression (6, 17–21).

The GRMD dog is an important large animal model of DMD (1, 2). Cardiac disease features in affected dogs that closely parallel those in boys include ECG abnormalities (22–24), initial lesion onset around adolescence (4, 5, 25), changes predominantly in the basolateral left ventricle (LV) (4, 5, 25, 26), and eventual progression to clinically evident dilated cardiomyopathy with reduced ejection fraction and heart failure (1, 2, 4, 5). Previous pathologic descriptive studies, including the first paper by Valentine et al. (25), established the basic features of GRMD cardiomyopathy (1, 25–29). However, these studies often focused on young dogs, and some assessed a limited number of cardiac anatomic sites. The Valentine study was comprehensive in that twenty four dogs were assessed, and 13 to 17 cardiac sections were sampled in each of them, but 19 of the 24 dogs were <12 weeks old and had no detectable abnormalities. Accordingly, the study focused on only five dogs ranging from 6.5 months (m) to 6 years (y). All five dogs had lesions, establishing 6.5 m as the age of onset for pathologic changes. Based on a study of eight crossbred beagles (CXMDJ) with the GRMD mutation, Yugeta et al. (26) suggested that the pathologic onset might be delayed until 12 months. Their pathologic assessment included only four dogs >12 m of age, with the oldest being 21 m. No histopathologic lesions were seen in dogs <12 m, and only 3 of the 4 older dogs had changes. While the most recent descriptive study out of Brazil (29) included 18 GRMD dogs and also made the most concerted effort to differentiate lesions among age groups, only single sites from the LV and right ventricle (RV) were examined for each dog. Since all the dogs in the Brazil study purportedly died of heart failure, the pathologic findings across all age groups might be more typical of end-stage disease *vs*. the full spectrum of lesion development.

Women carriers heterozygous for *DMD* gene mutations are also at an increased risk for clinical heart disease (30–33) due to mosaic cardiac dystrophin expression (30). Canine GRMD carriers have an analogous mosaic cardiac dystrophin expression pattern (34) and lesions similar to affected dogs, including variable degrees of myocardial fibrosis, necrosis, mineralization, and fatty infiltration concentrated in the LV free wall (35).

Given the similarities between the DMD and GRMD cardiomyopathies, preclinical studies in affected dogs should inform the management of DMD patients. Pathologic studies typically serve as the gold standard for establishing disease progression and aid in selecting appropriate diagnostic and prognostic markers. We report a semi-quantitative natural history of histopathologic cardiomyopathy changes in a large cohort of GRMD dogs across a wide age range.

## Materials and Methods

### Animals

Care of the dogs in this study was governed by principles outlined in the Guide for the Care and Use of Laboratory Animals of the National Research Council and the protocols approved by the institutional animal care and use committee at Texas A&M University. Typically, in our colony, the carrier females are bred with semen from affected males, producing 25% each of normal males, carrier females, dystrophic hemizygous males, and dystrophic homozygous females.

Hearts were collected from thirty eight dogs, including 7 normal hounds (controls; 2–10 y; 5 female and 2 male), 5 GRMD carriers (3 m−4.5 y), and 26 GRMD-affected dogs [divided among four age groups: <6 m (five dogs), 6–12 m (seven dogs), 12–24 m (five dogs), and >24 m up to 76 m (nine dogs)]. The GRMD dogs were evenly split between hemizygous males and homozygous females.

### Hearts

We collected hearts from GRMD-affected dogs that died or were euthanized due to declining quality of life (defined as an inability to maintain sternal recumbence or progression to end-stage heart failure) or at terminal end-points for studies unrelated to cardiomyopathy. Of the GRMD dogs, only one was euthanized due to reasons related to intractable heart failure; four died or were euthanized for other reasons. Hearts from carrier dogs were collected at terminal end-points for studies unrelated to cardiomyopathy and from one dog (3 m) that died under anesthesia with no previous clinical symptoms of heart disease. Unaffected hearts were collected from an unrelated colony of working hound dogs euthanized for non-cardiac events.

In all hearts, samples from the LV and RV free walls were snap-frozen at necropsy and banked for potential gene expression and immunohistochemistry studies. The remaining whole heart was placed in formalin.

### Gross Pathology and Sectioning

**Figure 1:**
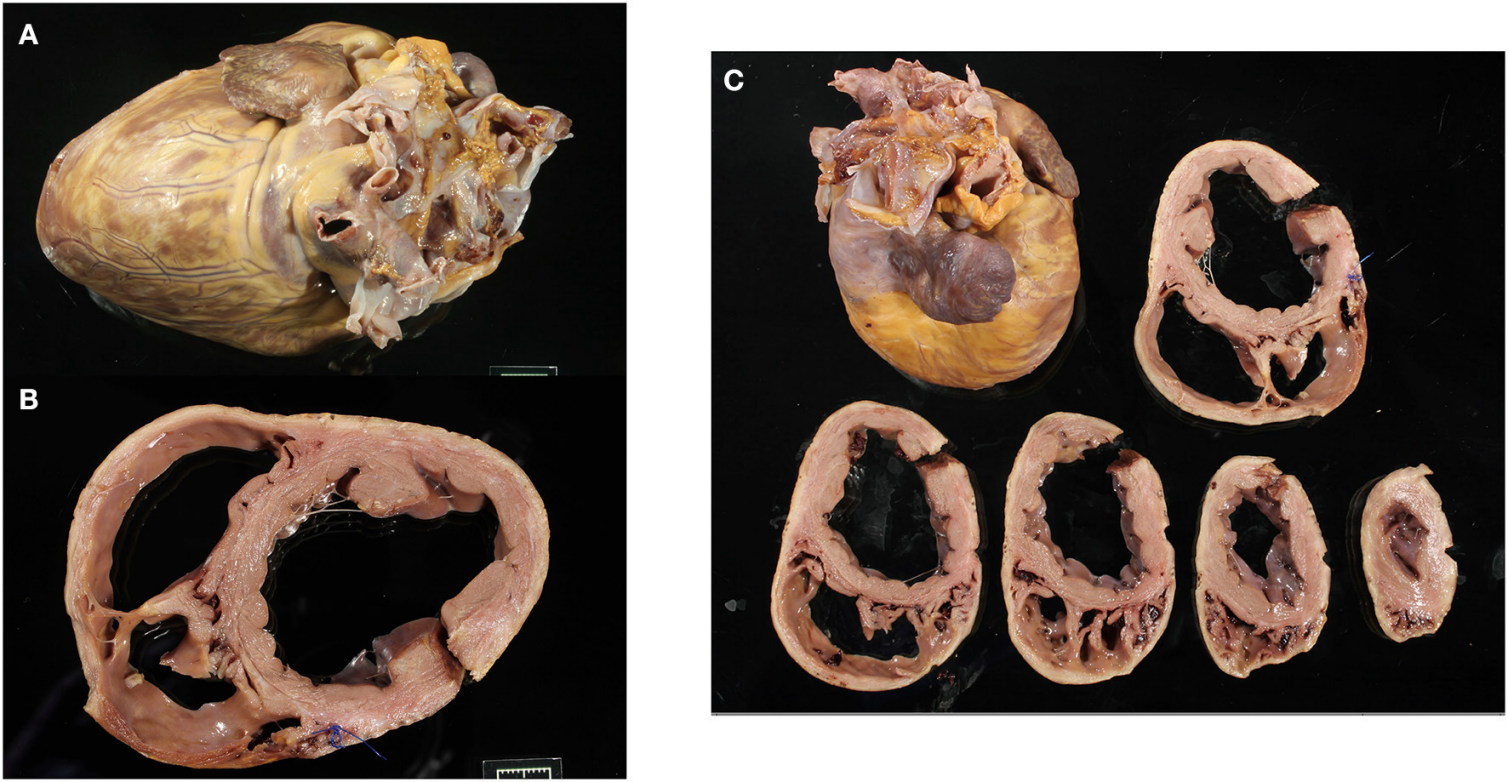
Representative gross images of a Golden Retriever muscular dystrophy heart showing the whole heart **(A)**, the basal cross-section with left ventricle and right ventricle dilation **(B)**, and all ventricular cross-sections **(C)**.

Fixed hearts were weighed and photographed in six views (cranial, caudal, lateral from left and right sides, base, and apex). Each heart was cross-sectioned through the ventricular short axis at 1-cm intervals to the level of the atrioventricular valves and photographed (Figure 1). The hearts were weighed, and the thicknesses of the fixed LV, RV, and interventricular septum were measured (Table 1 and Supplementary Table 1). The hearts were evaluated grossly and in the images for subjective dilation, pallor, and mineralization.

**Table 1:** Averages of gross heart measurements for Golden Retriever muscular dystrophy (GRMD), carrier, and normal dogs.

	**Averages (±SD)**								
**Group (*N*)**	**HW (g)**	**BW (kg)**	**LV (cm)**	**RV (cm)**	**Septum (cm)**	**HW/BW (g/kg)**	**RV/LV**	**LV/Sept**	**LV/HW (mm/g)**
All GRMD (26)	118.04 (±50.9)	15.43 (±7.47)	1.09 (±0.27)	0.46 (±0.14)	0.98 (±0.25)	8.74 (±5.33)	0.43 (±0.11)	1.15 (±0.33)	0.11 (±0.04)
GRMD >6 months (21)	135.14 (±41.1)^*^	17.4 (±6.98)^*^	1.16 (±0.25)^*^^‡^	0.5 (±0.13)^*^^†^	1.03 (±0.24)^*^	9.27 (±5.8)	0.44 (±0.12)	1.18 (±0.34)^‡^	0.09 (±0.02)
Carrier (5)	175.6 (±68.1)	15.32 (±3.22)^†^	1.38 (±0.16)^‡^^§^	0.66 (±0.05)^†‡^	1.22 (±0.17)^†^	12.32 (±6.44)	0.48 (±0.06)	1.14 (±0.12)^§^	0.09 (±0.05)
Normal (7)	210.83 (±30)^*^	25.09 (±0.9)^*^^†^	1.86 (±0.05)^*^^§^	0.9 (±0.14)^*^^‡^	1.91 (±0.05)^*^^†^	8.48 (±0.10)	0.49 (±0.04)	0.99 (±0.09)^‡^^§^	0.09 (±0)
**GRMD by age (** ***N*** **)**									
<6 months (5)	46.2 (±3.87)	7.16 (±1)	0.8 (±0.14)	0.3 (±0)	0.78 (±0.17)	6.52 (±0.62)	0.39 (±0.07)	1.05 (±0.21)	0.11 (±0.04)
6–12 months (7)	109.86 (±17.14)	17.67 (±7.12)	1.19 (±0.2)	0.57 (±0.12)	1.17 (±0.22)	6.90 (±1.88)	0.48 (±0.07)	1.02 (±0.11)	0.1 (±0.01)
>12–24 months (4)	94.75 (±8.1)	16.78 (±6.1)	0.95 (±0.21)	0.39 (±0.05)	0.98 (±0.04)	6.36 (±1.96)	0.42 (±0.06)	0.97 (±0.19)	0.1 (±0.02)
6–24 months (11)	104.36 (±16.24)	17.35 (±6.78)	1.10 (±0.23)	0.50 (±0.13)	1.10 (±0.20)	6.70 (±1.93)	0.46 (±0.07)	1.00 (±0.15)	0.11 (±0.01)
>24 months (10)	169 (±32.57)	17.46 (±7.2)	1.23 (±0.25)	0.5 (0±.12)	0.95 (±0.25)	12.09 (±7.16)	0.43 (±0.15)	1.37 (±0.39)	0.09 (±0.02)

^*^/^†^, p < 0.001;

*^‡^/^§^, p < 0.05*.

Each heart was systematically sectioned for histology. Seventeen sections of the LV (including septum) were taken to correspond with standard segmentation for advanced imaging (36). Additionally, four sections of the RV (anterior and inferior as available at the basal and mid-level) and the left and right atria were collected. The atrioventricular node was isolated by sectioning the septal junction of the right atrium below the foramen ovale and the non-coronary cusp of the aortic valve. The sinoatrial node was sectioned at the junction of the vena cava and the right atrium (37). Tissue cassettes containing the sections were photographed for reference, paraffin-embedded, and routinely processed. Slides were cut at 5-10 m thickness and stained with hematoxylin and eosin (H&E) and Masson's trichrome. All slides were digitally scanned at × 20 on a Hamamatsu NanoZoomer 2.0-HT whole-slide imager (Hamamatsu Corporation; Bridgewater, NJ, USA).

### Microscopic Pathology

Digital slide images were evaluated by a single pathologist (SS) using two methods to assess disease severity. Each section was given a semi-quantitative grade for approximate percentage of the cross-sectional area affected by histopathologic lesions, generally following the system described by Kane et al. (35) (0 = none, 1 = 1–10%, 2 = 11–20%, 3 = 21–30%, and 4 >30%) (Supplementary Figure 1). These scores were averaged across sections and groups for statistical comparisons (Supplementary Table 2). In a second method, specific histopathologic changes were scored as absent/present, and notations were made for the nature of some changes. The six assessed lesions included the following: fatty infiltration (absent/present), acute necrosis (absent/present), inflammatory infiltrates (none, primarily histiocytic, or primarily lymphoplasmacytic), mineralization (absent/present), Purkinje fiber vacuolation (positive if 50% or more of overall fiber cytoplasm was affected by vacuolation), and the nature of vascular changes (none, fibrosis, wall hypertrophy, and intimal hypertrophy).

**Figure 2:**
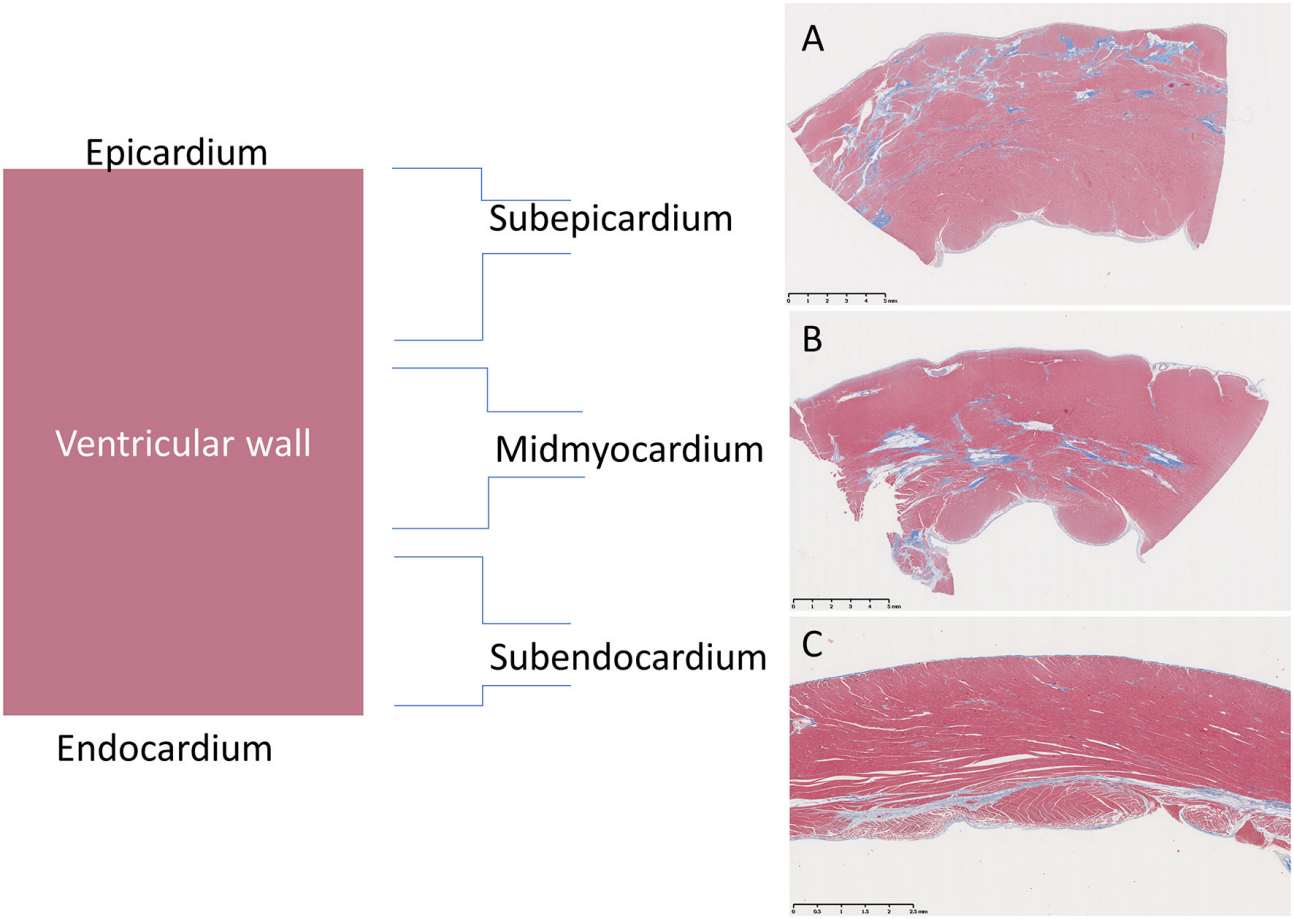
Representation of distributions of fibrotic lesions within the ventricular wall of Golden Retriever muscular dystrophy (GRMD) dogs using a trichrome stain which stains collagen blue and muscle fibers red. **(A)** The sub-epicardial myocardium (male, 3-years-old) is reportedly preferentially affected in GRMD dogs (pictured) and Duchenne muscular dystrophy boys (not pictured). **(B)** However, the mid-myocardium (male, 4-years-old) may predominate. **(C)** Subendocardial changes were rare and only seen in the right ventricle (female, 1-year-old).

The approximate percentage of examined sections from the whole heart with the six individual lesions mentioned above was semi-quantitatively scored using a modified “+” approach, whereby (−) = 0, (−/+) <0.5%, (+) = 0.5–20%, (++) = 20–30%, (+++) = 30–40%, and (++++) >40% (Tables 2, 3). Additionally, the primary lesion localization within each segment was classified as either subepicardial (outer 1/3), mid-myocardial (middle 1/3), subendocardial (inner 1/3), or panmyocardial (evenly distributed across the wall) (Figure 2).

**Table 2:** Averages of histologic changes for groups.

		**Frequency for individual lesions**					
**Group (*N*)**	**Semi-quantitative average lesion score**	**Fatty** **infiltration**	**Inflammation**	**Acute necrosis**	**Mineral**	**Vascular changes**	**Purkinje fiber vacuolation**
**GRMD age groups (** ***N*** **)**							
<6 months (5)	0.33	-	-	-	-	-/+	+
6–12 months (7)	0.85	+	+++	+	+++	++	++
>12–24 months (4)	0.73	+	++	+	+	+	+++
>2 years (10)	1.63	++++	++	-/+	+++	++	++
**Carrier (5)**	0.55	++++	+	-/+	+	+	+
**Normal (7)**	0.30	+	+	-/+	-	+	+

*The second column from the left depicts the semi-quantitative average score severity of all lesions taken together across left ventricle and right ventricle. The six columns to the right depict the percentage of all examined sections with the individual lesions listed: - = 0, -/+ <0.5, + = 0.5–20, ++ = 20–30, +++ = 30–40, ++++ >40*.

**Table 3:** Lesion frequency across the heart for individual dogs.

		**Frequency for individual lesions listed**					
**Age (months) and sex**	**Semi-quantitative lesion score**	**Fatty infiltration**	**Inflammation**	**Acute necrosis**	**Mineral**	**Vascular changes**	**Purkinje fiber vacuolation**
**GRMD**							
3 F	0.2	-	-	-	-	-	+
3 F	0.4	-	-	-	-	-	+++
3 F	0.58	-	-	-	-	-/+	-
3 M	0.16	-	-	-	-	-	-/+
3 M	0.22	-	-	-	-	-/+	+++
10 M	0.19	-/+	-/+	-	-	+	++++
11 F	1.05	++	++++	-/+	++++	+	-
11 M	0.3	-/+	+	-	-	+	++
12 F	1.47	++++	++++	-/+	++++	+++	+++
12 F	0.76	-	++++	++++	++++	++	-/+
12 F	1.39	-	++++	++	+++	+	+
12 M	0.81	-	++	+	+	++	++++
15 M	0.67	-	+	-	-/+	+	++
17 M	0	-	-	-	-	+	++++
21 F	1.29	++	++++	+	+	+	++
21 F	0.95	-/+	++	-/+	+	+	+++
28 F	1.93	+++	++	-/+	+++	+	-
36 M	0.95	++++	+++	-/+	++++	-/+	++++
38 M	1.19	++++	+	+	-	+++	++++
40 F	1.74	++++	+	+	++++	+	+
40 F	1.95	++++	-	-	+++	++++	-
40 M	1.86	++++	+	-	+	++	++
45 F	2.26	++++	+	-	++++	++++	+
51 M	2.38	++++	++++	-	+	+	++++
76 M	1.07	++++	++++	-	++++	+++^*^+	-
76 M	1	++++	+	-	+	+	++
**Carrier**							
3 F	0.13	-	-	-	-	-	-
12 F	0.05	+	-/+	-	++	+	+
46 F	1.4	++++	++++	+	+	++	-
56 F	0.37	++++	-	-	-	+	-
65 F	0.80	++++	+	-/+	-	++	+
**Normal**							
22 F	0.38	+	-	-/+	-	-	+
36 F	0	-	+	-	-	+	-/+
46 F	0.68	-/+	++++	-	-	-/+	-
61 M	0.14	-	-	-	-	+	+
75 F	0.35	+++	-/+	+	-	+	++
102 F	0.09	+	-	-	-	-/+	-
124 M	0.45	++	+	+	-	-	-

Trichrome slides were quantitatively analyzed for percentage fibrosis. The NDPI files for each slide were exported as a JPEG at × 5 magnification using the NDPI to OME-TIFF Converter v1.5 (https://matthias-baldauf.at/software/ndpi-converter/). These JPEGs were cropped in Graphic Converter (http://www.graphic-converter.net/) to remove duplicate tissue sections and large blood clots. The final JPEGS were analyzed in ImageJ using batch macros (Supplementary Data S1) to distinguish the percentage of blue staining from the total area with staining.

### Cardiac Magnetic Resonance Imaging

Some affected GRMD dogs in this study were part of a separate natural history imaging study (38) and had their cardiac function assessed prior to pathological collection. The cardiac magnetic resonance (CMR) scans were performed as previously described (38), using a 3-T MRI machine (Siemens 3T Magnetom Verio, Siemens Medical Solutions) while the dogs were under general anesthesia. To identify the fibrotic lesions, late gadolinium enhancement (LGE) and a semi-quantitative scoring method (from 0 to 2; none to marked enhancement) was applied to indicate the severity of fibrosis in each myocardial segment as previously described (38). The LV segmentation method in CMR was similar to the histologic segmentation, with both based on the same standard of 17 LV segments (36) and modified for canine heart. The LV lesion distribution was further compared between the CMR and pathologic assessments.

### Statistics

Descriptive statistics were calculated for each variable, including sex and age. Gross measurements were averaged for groups, and means were compared with *t*-tests. Two-sample *F*-test for variance was done to compare variability in heart weight (HW)/body weight (BW) ratios between groups. Sections of the heart were categorized into 25 regions for comparisons (as described above, 17 LV segments, 4 RV segments, the left and right atria, and the atrioventricular and sinoatrial nodes). One-way analysis of variance (ANOVA) was conducted by comparing the mean and standard deviation of fibrosis percentage and semi-quantitative lesion scores across all regions of the heart and comparing fibrosis percentage among GRMD, carrier, and normal specimens. Ordinal logistic regression was used to determine if the ordered independent variable, age, was associated with an increase in semi-quantitative cross-sectional lesion score. The average number of sections with each of the six individual lesions detailed above was calculated, and an ANOVA was used to compare the frequency of change among GRMD, carrier, and normal as well as between GRMD age groups for each feature. An independent-sample *t*-test comparing fibrosis percentage was calculated, stratified by the sex of the dog from which the specimen was obtained. An ordered logistic regression was run, comparing a rise in semi-quantitative lesion and vascular scores. Finally, Spearman's rank-order analysis was performed to evaluate the correlation between the LGE and pathological scores, and an ANOVA was performed to compare the mean value of fibrosis percentage and LGE scores within the LV sections across the hearts of seven dogs. Statistics were calculated using STATA 15 (College Station, TX, USA) and Microsoft Excel (Redmond, WA, USA).

## Results

### Gross Findings

No gross lesions were noted in any of the five affected dogs under 6 m of age. Older dogs had multiple lesions that varied in severity and distribution. Of the 10–12-m-old dogs, 5/7 had gross changes, including LV (3/7) or RV (1/7) pallor and streaking, and 3/7 had fibrotic or mineralized foci in the papillary muscle. Only 1/7 had clear concomitant LV and RV dilation on gross exam. In the four dogs 12–24 m old, one each had RV (1/4) or LV (1/4) dilation. All ten dogs >24 m had gross changes, including LV dilation (7/10); RV dilation (4/10); atrial dilation (2/10); thin ventricular walls (2/10); and pallor and streaking in the LV (6/10), RV (3/10), and septum (3/10) or pallor with mineralization in the papillary muscle (3/10). In carrier dogs, 4/5 (all >12 m) had gross changes, including thickening of the LV and septum (3/5), right auricle enlargement (1/5), and LV and septal pallor and streaking (1/5).

Furthermore, *t*-tests were used to compare mean gross measurements among groups (Table 1). For BW, HW, and ventricular wall measurement comparisons, the immature (<6 m) dogs were excluded, unless otherwise noted, since HW/BW and ventricular ratios differ even between normal immature and adult dogs (39). Based on BW, normal hounds were significantly heavier (25.09 ± 0.09 kg) than either the carrier (15.32 ± 3.22 kg) or adult GRMD dogs (17.4 ± 6.98 kg; *p* < 0.001 for both); the BW of carrier and adult GRMD dogs was not significantly different. Similarly, average HW differed among groups, with adult GRMD (135.14 ± 41.05 g) < carrier (175.6 ± 68.9 g) < normal (210.83 ± 0.05g) (Table 1), but the HW disparity was only significant (*p* < 0.001) between normal and GRMD. To better account for the effect of body size on heart weight, HW/BW ratios were calculated. The HW/BW ratio did not differ among GRMD (8.74 ± 5.33), carrier (12.32 ± 6.44), and normal dogs (8.48 ± 0.10) when comparing all ages or when comparing only dogs >6 m (9.27 ± 5.80) (Table 1), suggesting that the lower HW in GRMD dogs was due to their smaller stature. The results for individual dogs are included in (Supplementary Table 1).

A two-sample *F*-test for variance was done to compare the variability in HW/BW ratios in the dogs. Interestingly, GRMD dogs >24 m and carriers >12 m had a significantly greater variance in the HW/BW ratios than either normal dogs or GRMD dogs <24 m, reflecting the wide phenotypic variation and disease progression in older dogs. In the GRMD dogs >24 m, the HW/BW ratio ranged from 5.85 to 31.4 g/kg (Supplementary Table 1), with four dogs falling well-above the previously reported range of 4.53 to 10.04 g/kg in large adult dogs (39, 40). Similarly, the 3 carrier dogs >24 m (28–76 m) had higher HW/BW ratios of 13.5 to 23.1 g/kg. The BW did not differ between GRMD dogs >24 m *vs*. those <24 m of age, but the HW was significantly higher in the older group (169 ± 32.6 *vs*. 104.4 ± 16.2 g; *p* < 0.001). This suggests that increased the HW/BW ratio is driven by a relative increase in HW in the older dogs.

Comparing the measurements among groups, the average LV thickness varied significantly: GRMD (1.09 ± 0.27 cm) < carrier (1.38 ± 0.16 cm) < normal (1.86 ± 0.05 cm) (*p* < 0.05) (Table 1), but the ratio of RV to LV thickness did not differ. Of the five carriers, four had subjective wall and papillary muscle thickening relative to the heart size on gross exam. However, when correcting the LV thickness for overall HW, the groups were not significantly different (Table 1), which suggests that hearts with thicker walls were proportionally heavier.

### Semi-quantitative Lesion Scoring in GRMD Dogs

Using both H&E- and trichrome-stained images, the sections were scored semi-quantitatively based on the total cross-sectional area percentage, as generally described by Kane et al. (35) (0 = none, 1 = 1–10%, 2 = 11–20%, 3 = 21–30%, and 4 >30%). The heart sections were categorized into 25 regions for comparisons, as described above. Among GRMD dogs, the scores for individual sections varied from 0.7 (several sites) to 1.4 (basal anterolateral). The scores for carriers were lower, varying from 0.3 to 1.2, and the sites with greater/lesser involvement did not correspond to the GRMD group. Normal dogs had lower scores, ranging from 0.1 to 0.7. The overall average cross-sectional lesion area was higher in the GRMD *vs*. normal dogs (1.02 *vs*. 0.3; *p* < 0.001). While the average score for carriers (0.6) was intermediate, it was not significantly different from either GRMD or normal dogs. On evaluation of one-way ANOVA comparing the average cross-sectional area score among sections, no section had a significantly higher or lower cross-sectional area score. The values for individual dogs and section averages are shown in (Supplementary Table 2).

Ordinal logistic regression was used to determine if the ordered independent variable, age, was associated with an increase in semi-quantitative lesion score. The scores for each section showed a highly statistically significant (*p* < 0.001) correlation with age, consistent with disease progression over time.

#### Fatty Infiltration

**Figure 3:**
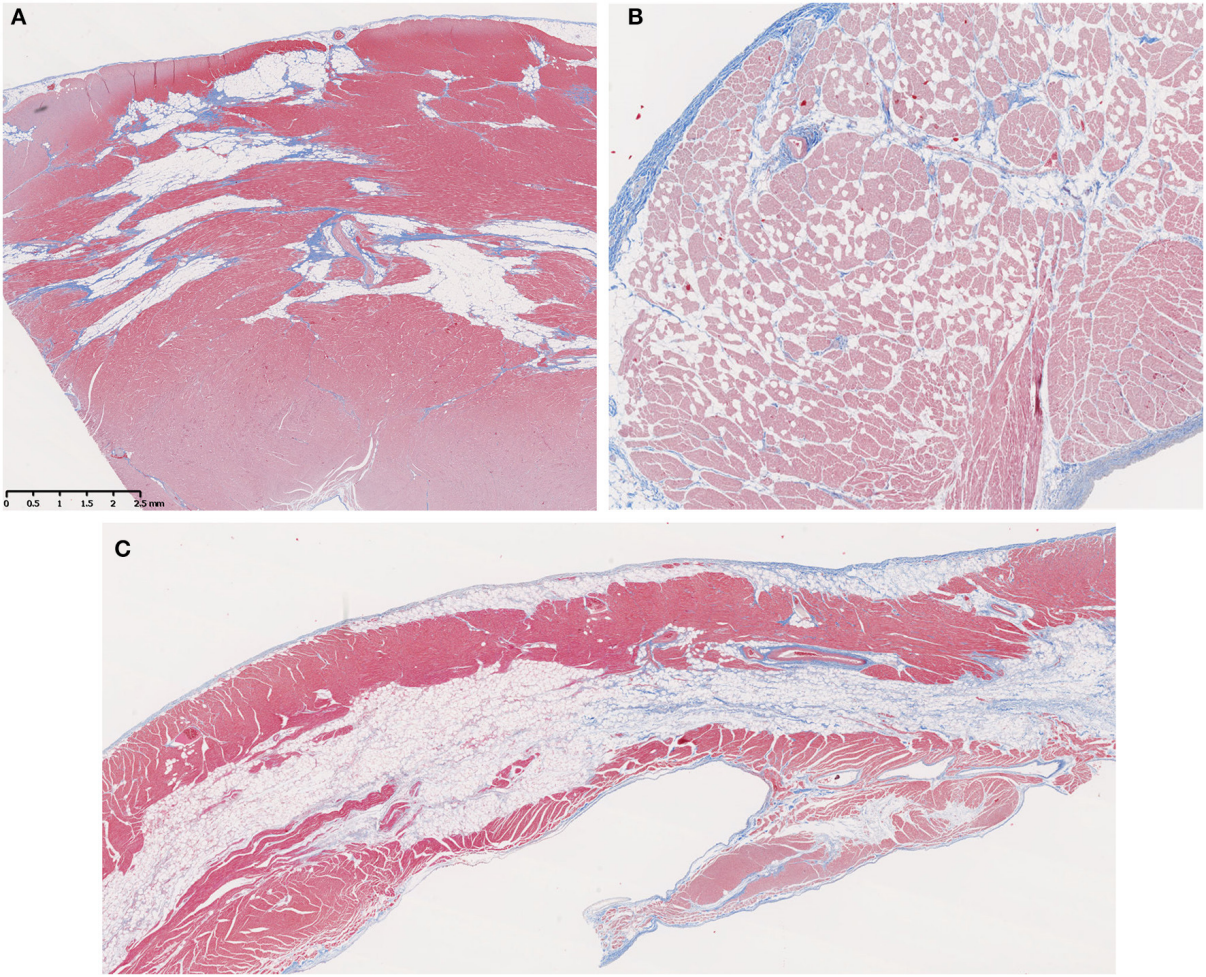
Fatty infiltration was a prominent feature in older Golden Retriever muscular dystrophy (GRMD) dogs. **(A)** A section of left ventricle free wall from a 3-years-old female GRMD dog shows large bands of fat (non-staining vacuolated tissue) replacing normal myocardial muscle (red) and predominating over fibrosis (blue). **(B)** In some cases, fatty infiltration was more diffuse with individual or small clusters of fat cells scattered throughout the myocardium (male, 1-year-old). **(C)** In several dogs, the RV showed striking mid-myocardial replacement by bands of fat with minimal fibrotic tissue (male, 3.5-years-old). In these trichrome-stained sections, muscle fibers = red, collagen = blue, and fat cells = non-staining vacuolated tissue; × 10 original magnification.

Fatty infiltration (Figures 3A,B) was frequently seen in the hearts from older GRMD and carrier dogs (Table 2). While the percentage of affected sections in each normal heart ranged from 0 to 30%, GRMD and carrier dogs >24 m all had >50% of sections showing fatty infiltration and degeneration. In GRMD dogs >24 m, 195/232 (84%) of the examined sections had fatty change. A similar percentage of sections (56/76, 74%) in carriers also showed fatty degeneration, while only 18/149 (12%) of normal sections had this lesion. However, only the differences between GRMD and normal dogs were significant (*p* < 0.01), probably due to the relatively low number of carriers.

Fatty infiltration and degeneration increased with age. None was seen in any dog <6 m. The GRMD dogs >24 m had a significantly higher proportion of sections with fatty infiltration (84.05%) than any of the younger GRMD groups (0%, 6 m; 14.75%, 6–12 m; 16.95%, >12–24 m; *p* < 0.001) (Table 2). The same was likely true for carriers, in which only two sections in one dog <12 m had fatty infiltration, although the numbers were too small to determine significance. No normal dog was <22 m; the oldest three dogs had the highest number of affected sections in this group (Table 3).

Fatty replacement of the mid-myocardium was a prominent feature in the RV of some GRMD dogs (Figure 3C), with only small bands of muscle remaining on either side of a central band of adipose tissue. This pattern was not observed in the left ventricle, where patchy areas of fat tended to surround vessels and be mixed with fibrosis (Figure 3A).

#### Acute Necrosis

**Figure 4:**
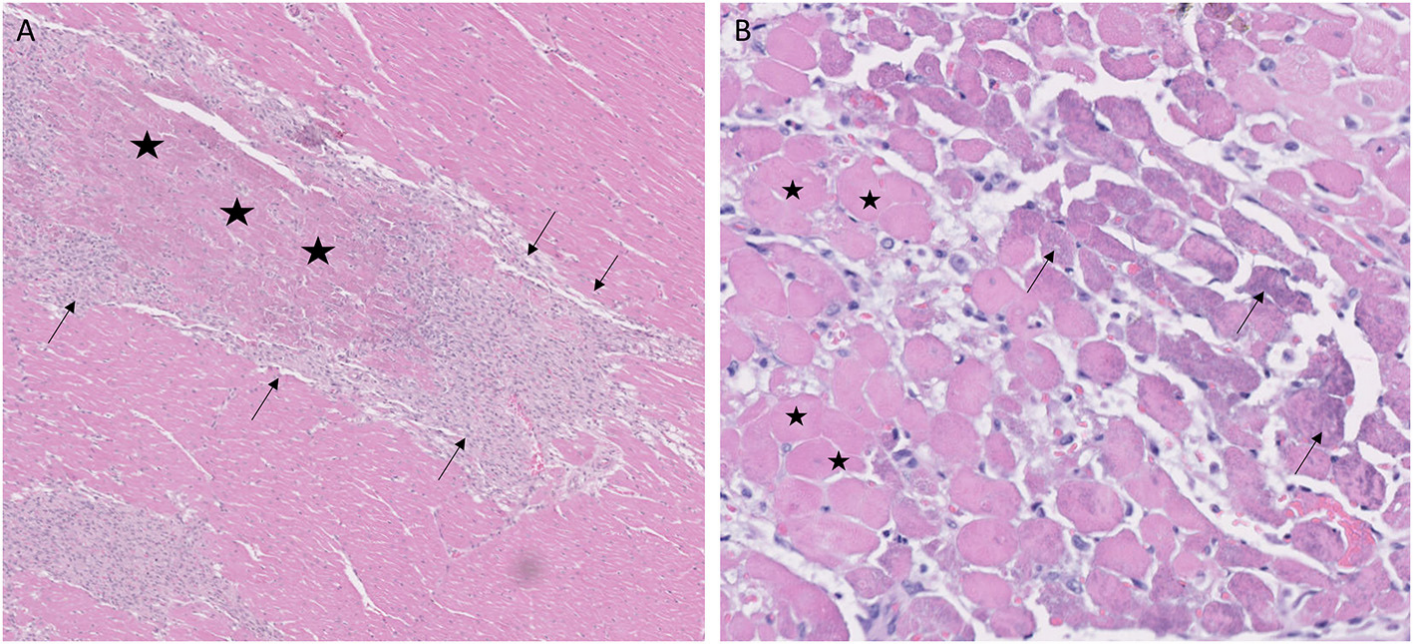
Coagulative myocardial necrosis was a feature in some Golden Retriever muscular dystrophy hearts. **(A)** Areas of infarct-like coagulative necrosis characterized by loss of cytoplasmic detail and maintenance of tissue architecture (stars) are surrounded by proliferating macrophages and pericytes (arrows); H&E, × 5 magnification. **(B)** Acute coagulative necrosis with loss of nuclei and hypereosinophilic cytoplasm (representative cells with stars); cells on the margins of lesions have granular basophilic mineralization (arrows). 1-year-old female, H&E, × 20 magnification.

Of the 26 GRMD dogs, 0/5 <6 m (0%), 5/7 6–12 m (71%), 2/4 12–24 m (50%), and 4/10 >24 m (40%) had at least one section with acute coagulative necrosis (Figure 4). Nearly half the number of GRMD dogs with this change (5/11) had three or more sections with acute necrosis. Over half the number of dogs with acute necrosis (7/11) were 6–24 m, pointing to the relatively early onset of this lesion. Acute necrosis was found at least once in the 17 LV and 4 RV sections that were examined in each dog, with no one heart section having a higher likelihood of lesions. Acute necrosis was not found in the atria. Incidentally, 3/7 normal dogs and 2/5 carrier dogs, each >24 m, also had at least one, but no more than three, sections with acute myocardial necrosis (Table 3).

One 12-m-old female GRMD dog had markedly more affected sections (14/27 heart segments) with acute myocardial necrosis. This dog had an incident of marked abdominal breathing and dark urine of undetermined cause following anesthesia for wound treatment at ~6 m of age. No hyperthermia was documented, and blood oxygenation was maintained in the normal range. As part of a non-cardiac study, she was euthanized while under anesthesia at 12 m of age.

#### Inflammation

Inflammatory cells were seen in all disease groups and did not differ among ages, even if the youngest dogs were excluded (Table 2). While the overall scores for inflammation among normal, GRMD, and carrier dogs did not differ, there were differences in the cell types. Normal dogs had lymphoplasmacytic infiltrates, with one 46-m-old dog having infiltrates in almost every examined section. Although normal dogs were euthanized for non-cardiac lesions, two dogs initially examined for the study were excluded due to chronic Chagas disease (*Trypanosoma cruzi*) detected histologically and by PCR. *T*. *cruzi* was not detected in the remaining dogs, but exposure with associated lesions may explain the unexpected inflammation in some dogs from this group. Conversely, carriers and GRMD dogs had few sections with lymphoplasmacytic infiltration (four in all the examined dogs), with predominantly histiocytic inflammation. Histiocytic inflammation is typical secondary to degeneration and acute necrosis, initially occurring within 48 h and peaking at around 6 to 10 days, with slow resolution as the tissue remodels (41, 42).

Interestingly, when comparing the age groups, dogs <6 m had significantly fewer sections with inflammation (no inflammation) than 10–12- and >24-m-old dogs (*p* < 0.05 and *p* < 0.01) but were not significantly different from the small group of 12–24-m-old dogs. Dogs of 10–12 m trended toward more inflamed sections than 12–24-m-old dogs (*p* = 0.054), while dogs >24 m had significantly more affected sections than 12–24-m-old dogs (*p* < 0.05). However, the 10–12-m- and >24-m-old dogs did not differ.

#### Mineralization

No mineral was noted in the hearts from normal dogs. All GRMD dogs >10 m had mineral in at least 1 section, with the exception of a 17-m-old male that had few lesions of any type (Table 3). The average number of sections containing mineral differed significantly (*p* < 0.05) among the GRMD age groups (<6 m: 0 sections, no detectable mineral; 6–12 m: 8.4 sections; 12–24 m: 2 sections; >24 m: 8.8 sections). The low numbers in the 12–24-m group likely reflect the relatively mild lesions in this age group in our study, as the degree of mineralization has previously been shown to decline with age (25). Two of five carriers also had mineral in at least one section.

#### Purkinje Fibers

**Figure 5:**
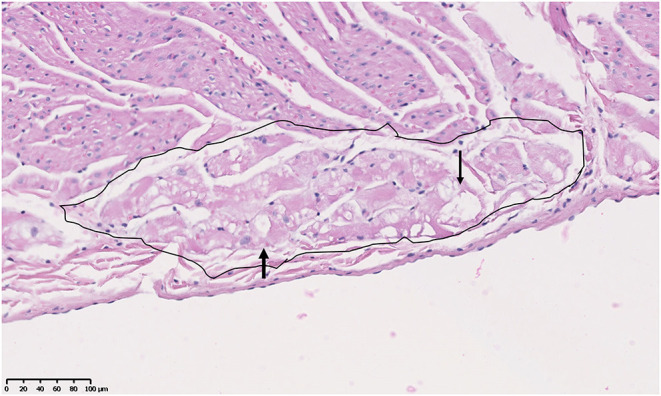
Purkinje fiber (circled area) vacuolation (clear spaces indicated by arrowheads) in the heart of an 18-month-old male Golden Retriever muscular dystrophy dog. H&E, × 20 magnification.

Previous studies described but did not score Purkinje fiber vacuolation starting at under 6 m in the GRMD (CXMDJ) heart (27). Degenerative changes have also been described in Purkinje fibers of DMD boys (43). We scored Purkinje fiber vacuolation as present if vacuoles affected 50% or more of overall fiber cytoplasm (Figure 5). Of the 26 GRMD dogs, 4/5 (80%) <6 m, 6/7 (86%) 6–12 m, 4/4 (100%) 12–24 m, and 7/10 (70%) >24 m had at least one segment with vacuolation. The GRMD dogs had significantly increased fiber vacuolation (average, 6.9 affected sections; range, 0–19) compared to both carriers (1.4 sections; *p* < 0.01) and normal dogs (1.6 sections; *p* < 0.01) (Tables 2, 3). Purkinje fiber vacuolation did not differ between carrier and normal dogs nor among age groups in the GRMD cohort. Unlike the previous CXMDJ study, some GRMD dogs had no Purkinje fiber vacuolation detected. Moreover, vacuolation was not correlated with age nor with either semi-quantitative or quantitative lesion scores. Indeed, vacuolation was marked in some of the youngest dogs that otherwise had no lesions. The examination of H&E slides did not show an appreciable visual difference in the vacuoles among groups. The sinoatrial and atrioventricular nodes did not have observable abnormalities with H&E or trichrome staining.

#### Vascular Changes

**Figure 6:**
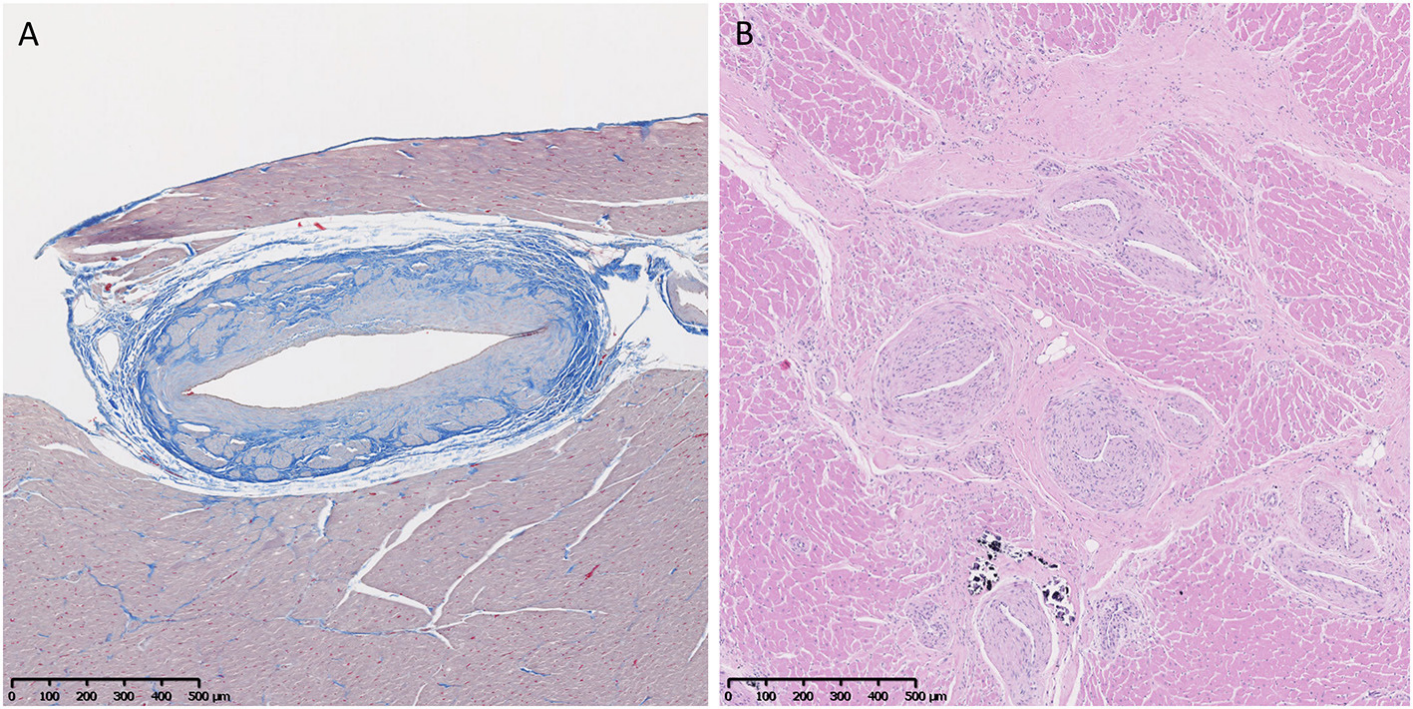
**(A)** Increased fibrous tissue (blue) in an arteriole of a normal 3-year-old female. Trichrome, × 5 magnification. **(B)** Marked medial and intimal hypertrophy and vascular proliferation in arterioles in the heart of a 6-year-old male Golden Retriever muscular dystrophy dog. H&E, × 5 magnification.

Vascular changes of fibrosis and medial hypertrophy (Figure 6) were evaluated. Hearts from GRMD dogs <6 m had only rare changes. Those from adult (>6 m) GRMD dogs had a significantly greater frequency of vascular wall hypertrophy than normal dogs (5.2 *vs*. 1.7 sections; *p* < 0.01). Although low numbers prevented the detection of a difference between normal and carrier dogs, two of the carriers had hypertrophy in twice as many sections as the highest normal dogs.

Ordered logistic regression comparing a rise in the semi-quantitative cross-sectional score (which did not include vascular changes) and vascular scores in GRMD dogs showed a highly significant association between arteriolar hypertrophy and a rise in semi-quantitative severity score. For every 1-point increase in the vascular score, there was a 0.32 rise in the semi-quantitative severity score (*z*-score = 3.91, *p* < 0.001). Although correlated, it is not clear if there is a causative association or if both represent a cumulative increase in degenerative changes with age.

Additionally, aortic mineralization was a prominent feature in GRMD dogs, with 11/26 having prominent mineralization in the aorta: 0/5 <6 m, 4/7 6–12 m, 1/4 12–24 m, and 6/10 >24 m of age. Aortic mineralization was not noted in the carriers, while 2/7 of the oldest normal hounds had much smaller mineral deposits in the aortic wall.

Although endothelial hypertrophy was described by Valentine et al. in one dog, it was not detected in this study.

### Quantitative Fibrosis

An analysis of variance was performed to assess the relationship between disease status and the percentage of cross-sectional area fibrosis as measured by trichrome staining. Data from all age groups, including more mildly affected dogs <6 m, were included. All three groups were highly significantly different (*p* < 0.001), with carriers having greater fibrosis (mean: 9.82 ± 1.01% blue staining) compared to GRMD (7.84 ± 0.25%), while normal dogs (4.35 ± 0.26%) had the lowest percentage. An independent *t*-test indicated no difference between the sexes in fibrosis.

Additionally, the degree of fibrosis staining differed significantly among GRMD age groups as determined by one-way ANOVA (*f* score = 15.01, *p* < 0.001). The Tukey *post-hoc* test revealed that the mean value at 6–12 m (5.38 ± 3.54%) was significantly lower than all other groups (*p* < 0.05) and that the 12–24-m group had the highest degree of fibrosis (9.80 ± 8.31%), being higher than even the >24-m group (8.28 ± 5.23%; *p* < 0.05). Lower values in the oldest dogs could reflect greater fat deposits, which are not detected by this method, with proportionally lower fibrosis.

One-way ANOVA showed a statistically significant difference between groups (*f* score = 7.64, *p* < 0.001) for the degree of fibrosis staining across the 17 LV and 4 RV sections from GRMD dogs. The Tukey *post-hoc* test revealed that the RV sections had the highest levels of fibrosis compared to the other sections but were similar among themselves (*p* < 0.001). The mid-LV anterolateral section had significantly lower fibrosis than the other sections.

### Localization of Lesions Within the Wall

Previous studies of GRMD (25) and DMD (4) describe a diffuse cardiomyopathy with sometimes prominent sub-epicardial localization of fibrosis and other lesions. We scored the predominant localization in each section as primarily sub-epicardial, mid-myocardial, sub-endocardial, or transmural (even distribution across the myocardial wall) (Figure 2). Consistent with the previous observations, degenerative lesions were initially concentrated in the outer half of the myocardium (the sub-epicardium and mid-myocardium or the RV portion of the septum wall). In older dogs, more sections had transmural lesions as might be expected in a progressive disease. In general, the basal LV sections had a greater degree of outer (or sub-epicardial) lesions, while the RV and more apical portions had a more diffuse/transmural distribution overall or, as described above, a prominent mid-myocardial distribution, particularly in the more apical RV.

### Correlation With LGE in Cardiac MRI

Correlating specific histologic findings with diagnostic imaging is the ultimate goal to better track cardiomyopathy progression. CMR with late gadolinium enhancement is a common modality for the clinical assessment of cardiomyopathy in GRMD and DMD (44). Eight GRMD dogs in this study were part of our cardiac imaging natural history study (38), and seven of them had sufficient LGE imaging scans within 7 days prior to heart collections, allowing a retrospective comparison with the pathologic results (the preliminary data of one dog was previously reported, including a representative visual comparison of gross, MRI, and histologic images) (38). In order to evaluate the correlation between LGE and the pathologic assessment within each section of the heart across the seven dogs, Spearman's rank-order analysis was performed. Observations without a pair between approaches were removed from analysis. This non-parametric test revealed a highly significant (*p* < 0.0001) monotonic relationship between these two methods, with a moderately positive relationship coefficient of 0.44. The LGE and semi-quantitative pathology scores for each section increased together, showing a consistency between the imaging and pathologic assessments. Fibrosis percentage was also compared to the three LGE categories across the same sections (*N* = 107) by performing a one-way ANOVA. The one-way ANOVA did not produce statistically significant results (*p* = 0.493). While there was a slight increase in fibrosis percentage in the highest LGE category, this is likely due to an increase in findings within this category compared to other groups. Based on this analysis, there is not a clear correlation between these variables.

## Discussion

Myocardial disease in DMD is classically defined as dilated cardiomyopathy with more pronounced LV *vs*. RV lesions and functional changes (4–6). Prior studies of the GRMD cardiomyopathy have been limited by either the number of dogs studied or the anatomical sections assessed (25, 26). This paper describes the gross and histologic lesions across the heart in a large cohort of GRMD dogs ranging from 3 to 76 m in age. Like the previous more limited studies, we did not see an overall increase in HW/BW ratio typically associated with hypertrophic cardiomyopathy. However, the HW/BW ratios of some GRMD and carrier dogs were 3 to 4 times higher than the normal range (39, 40). While decreased BW due to muscle atrophy in GRMD dogs could contribute to increased HW/BW ratio, there was little difference in BW among the groups. On the other hand, HW increased in the oldest GRMD and carrier dogs. Moreover, HW was not directly correlated with age in the adult dogs. Taken together, these findings suggest that elevated HW/BW ratios in affected and carrier hearts occurred due to eccentric cardiac hypertrophy as previously reported in our dogs (38) and seen in canine dilated cardiomyopathy (45), mdx mice (46), and in some studies of DMD carriers (47) and DMD boys (48, 49).

Fibrosis is the principal pathological change in DMD cardiomyopathy, occurring initially in the posterobasal portion of the left ventricular free wall and extending to the outer third and ultimately the entire left ventricle and septum (4, 22). The right ventricle may be dilated but, generally, is free of fibrosis. The GRMD dogs of this study also had pronounced LV lesions, but in contrast to DMD, the RV was also heavily affected, although often with more fatty than fibrotic changes. The cardiac lesions in DMD are consistently more pronounced in the subepicardium, with sparing of muscle fibers closest to the left ventricular chamber. This was also the case in the GRMD dogs studied here, but we did not detect the distinct posterobasal concentration seen with early DMD lesions, instead finding more diffuse changes. Notably, involvement of the papillary muscles and posterobasal area accounts for mitral valve regurgitation in DMD (50). The papillary muscles in GRMD dogs were also highly affected, although we were unable to link this to mitral valve dysfunction.

Similar to other studies (25, 26, 29) that assessed specific histopathologic changes, lesion severity tended to increase with age. Despite this general trend, some older dogs had few, if any, lesions (Table 3), in keeping with the marked phenotypic variation seen in the DMD cardiomyopathy (51, 52). Carrier hearts had lower levels of involvement, and normal dogs had the lowest scores. The lack of regional associations in our study may have occurred because the dogs were assessed at a single time point rather than longitudinally. The fact that many of our dogs were older and had lesions in all examined sections and that there were relatively few dogs in the potentially critical 12–24-m age range group may also have prevented the detection of regional differences in lesion development. With that said, our study had a distinct advantage in that hearts from dogs across many ages and stages of disease progression were included, while in DMD, routine cardiac biopsy is not commonly done, and autopsies are also uncommon, potentially causing the lesions outside of chronic fibrosis to be missed.

In quantifying fibrosis using the trichrome stain in adult (>6 m) dogs, those at 6–12 m had significantly less fibrosis than either of the two older groups. Unexpectedly, the dogs >24 m had less quantitative collagen staining than those at 12–24 m, even though half of these younger dogs had relatively mild lesions. The proportionally lower percentage of collagen staining may have occurred because lesions, such as fatty infiltration, non-compact fibrosis, and mineralization, are not stained by the methods used, and fat, in particular, was increased in the older dogs. Importantly, this demonstrates that methods aimed solely at fibrosis detection may underestimate phenotypic severity in older GRMD dogs. Semi-quantitative scoring of total cross-sectional lesion area and the percentages of sections showing various lesions more clearly demonstrated a strong correlation with age and disease progression. This scoring also highlighted the large phenotypic variation of cardiac lesions in GRMD dogs, similar to the variation in skeletal muscle disease (2). In particular, one 17-m-old GRMD male had no cardiac lesions. All semiquantitative scoring tasks were done by a single pathologist in this case. This method would likely have a higher inter-operator variability, making it unreliable for comparing results between studies without more robust agreement on scoring. Having multiple interpreters for each slide with a consensus score would likely improve the result reproducibility.

Even more than fibrosis, fatty infiltration was a feature of cardiomyopathy progression in older GRMD dogs. While some fatty infiltration may be seen with normal aging, this change was particularly pronounced in the GRMD dogs, as is seen with other chronic canine cardiomyopathies (45, 53–56). Lipomatous (fatty) metaplasia is also seen with chronic degenerative conditions in the hearts of dogs and humans, most frequently with chronic ischemic cardiomyopathy and ischemic scar replacement (57, 58), with an associated increased propensity for ventricular tachycardia and arrhythmias (53, 59). Although we cannot rule out a specific response to dystrophin deficiency, the occurrence of a similar fatty change in a variety of diseases suggests it is a general reaction to chronic myocardial degeneration and remodeling.

Interestingly, coagulative necrosis was seen in some GRMD dogs in the absence of vascular thrombosis. The severity of these changes correlated with the degree of vascular thickening. Given that the coronary vascular supply to the ventricles differs between dog and man (60), it is difficult to draw inferences regarding DMD cardiac disease pathogenesis. However, we have previously seen a syndrome of acute myocardial infarction in GRMD dogs (61) that appears analogous to a condition in DMD (62). This has led to a speculation that dystrophin-deficient cardiomyocytes are operating in a state of functional hypoxia (61, 63, 64) and that a dystrophin-deficient muscle has reduced capacity to compensate for increased metabolic demands (65, 66). Coagulative necrosis is associated with anoxia/hypoxia, occurring often subsequent to ischemia, infarction, or toxicosis. Lesions are detected histologically at ~12–24 h post-event, become infiltrated by sheets of macrophages within 48–72 h, and contain foci of interstitial cell and vascular proliferation by 10 days to 6 weeks (41, 42). We found each of these lesions in all GRMD age groups older than 6 m, in keeping with the full range of cardiac necrosis and healing described by Malvestio et al. (29). Considering that vascular thrombosis is not a feature of DMD/GRMD, these changes more likely occur secondary to non-occlusive hypoxia. Extending the potential significance of vascular/hypoxic disease in GRMD cardiomyopathy, we also found a positive correlation between arteriolar hypertrophy and the most severe semi-quantitative cross-sectional lesion scores. Dystrophin-deficient mdx mice also have enhanced neointimal formation, with wall thickening and narrow lumens due to vascular smooth muscle proliferation (67). In principle, these changes could occur due to chronic degenerative lesions in dystrophin-deficient smooth muscle and might be a factor in the acute myocardial syndrome seen in DMD/GRMD. While increased wall thickness and connective tissue are a reported aging change in the cardiac arterioles of senescent dogs (68), the normal dogs in this study had significantly less smooth muscle hypertrophy than GRMD dogs, indicating that this change is part of disease progression.

Dystrophin deficiency in vascular smooth muscle in both mdx mice and GRMD dogs has been shown to have functional consequences, including altered sympathetic vasoregulation and reduced attenuation of vasoconstriction during contraction (67, 69–72). This is partly due to loss of sarcolemmal nNOS in skeletal and cardiac muscle, leading to decreased nitric oxide-induced vasodilation. However, the selective expression of dystrophin in vascular smooth muscle partially rectified the vasoregulatory responses (72) in mdx mice, suggesting that smooth muscle dystrophin may have a primary functional role in vasoregulation. In combination with the types of degeneration seen in the hearts of GRMD dogs of our study, these vascular changes could support the “two hits” hypothesis for lesions in dystrophin deficiency (73), with functional ischemia related to altered vasoregulation combined with increased susceptibility to metabolic stress leading to impaired cardiomyocyte survival.

Stereotypical ECG abnormalities are common in DMD and GRMD patients. The degenerative changes described in the Purkinje fibers of affected boys (43) and dogs (27) have been proposed as a possible contributing factor. Urasawa et al. (27) described marked abnormal vacuolation in Purkinje fibers associated with ultrastructural degenerative changes in young, crossbred beagles with the GRMD mutation (CXMDJ). We also detected increased Purkinje fiber vacuolation, starting with the youngest GRMD dogs, though this lesion was not present in all GRMD dogs, and normal dogs also had Purkinje fiber vacuolation. Interestingly, the carrier dogs of this study did not have Purkinje fiber vacuolation, even though they also have conduction abnormalities (35). Additionally, the degree of vacuolation did not correlate with cross-sectional lesion severity in our dogs. Due to the retrospective nature of this study, ECG was not available for correlation of vacuolar severity with conduction differences or arrhythmia.

The pattern of CMR LGE changes in DMD patients tends to parallel the distribution of histopathologic changes, beginning in the LV posterior basal and subepicardial wall (44). Although our LGE imaging quality was insufficient to localize lesions to the subepicardial region, the pathological results were consistent with the LGE findings. Moreover, the semi-quantitative assessments for histopathologic lesion score and LGE severity were strongly correlated, further validating the association between our pathologic and CMR findings. Conversely, quantitative fibrosis percentage was not correlated with LGE. As with the Purkinje fiber changes, we did not have an opportunity to determine whether there was an association between ECG findings, such as the occurrence of deep Q waves, with septal fibrosis. This would require a detailed longitudinal prospective study.

While the data reported here substantially extended prior pathologic studies of GRMD cardiomyopathy, limitations included the inability to perfectly age-match dogs across disease groups, low number of carrier dogs, variation in phenotypic severity, and necessity for using normal control hearts from dogs outside the colony. Heart availability and age across disease groups were limited by the availability of dogs dying for other reasons. Since carrier and normal dogs are infrequently euthanized at young ages, these groups, in particular, lacked exact-age-matched controls. Here, we assessed a separate cohort of hound dogs outside our colony. Although minimal differences would be expected between hearts from normal dogs within and outside the colony, variable housing and management could have altered the cardiac phenotype—for instance, two dogs were excluded due to PCR-confirmed Chagas's disease, suggesting that all the normal dogs may have been exposed to infections not present in our colony. That may also explain the unexpected lymphoplasmacytic inflammation in some hearts from this group.

Like previous studies, cardiac pathology was limited or absent in our GRMD dogs <6 m, and changes would likewise not be expected in normal dogs, so the inability to compare directly likely had a minimal impact. Carrier dogs that are not used for breeding are routinely adopted out of the colony, limiting our access to tissue from this group. The lower numbers hindered our ability to detect significance between this disease group and the GRMD and normal dogs; however, the trends are intriguing, and more studies are warranted.

The conclusions drawn from non-longitudinal studies are also always limited by the phenotypic variation in disease expression among dogs. In our cohort, we had a low number of dogs and a high degree of phenotypic variation in the seemingly critical 6–12-m age range, while the 12–24-m group seemed to have an overall milder severity, including one dog with no lesions. This likely muddled the differences between the age groups. The mild to non-detectable disease in dogs of this age that had not had any cardiac-specific treatments highlights the importance of not relying on a single animal to determine treatment effects.

This study provides the largest comprehensive gross pathologic and histopathologic review of GRMD cardiomyopathy to date. Our findings highlight the differences in disease severity and suggest that semi-quantitative scoring is preferable to fibrosis quantification for assessing the overall lesion severity. The disease is universally progressive, but due to the difficulty in detecting early lesions pre-mortem, there is still debate over the sequence of cardiac lesion progression in GRMD and, by extension, DMD. Based on previously published findings and the results reported here, we suggest that two interacting factors are involved. Underlying metabolic deficiencies in cardiac muscle, such as calcium dysregulation, membrane fragility, poor response to hypoxia, and mitochondrial abnormalities, place the fibers under a constant metabolic strain. Over time, this may lead to individual fiber necrosis, mineralization, and dropout, with fatty transition in some fibers, particularly in areas of strain (i.e., basoinferior LV wall). Additionally, episodic stress in the face of the vascular changes we have described could produce non-thrombotic infarction and loss of larger foci of myocardium compatible with large acute/subacute lesions in some dogs. Sequential episodes of damage may result in a stairstep cycle of damage, repair, and stabilization, with increasing strain, fatty metaplasia, and decreasing reserve over time.

## Data Availability Statement

The raw data supporting the conclusions of this article will be made available by the authors, without undue reservation.

## Ethics Statement

The animal study was reviewed and approved by the Institutional Animal Care and Use Committee at Texas A&M University.

## Author Contributions

SS, JK, BW, and L-JG contributed to the conception and design of the study including sample collection protocols. SS and BW contributed to the interpretation of histologic changes. SS developed the interpretation categories, collected the hearts and samples, wrote the first draft and major revisions of the paper, and scanned and read the slides. SF developed the Image J macro. SF and SS collected the image J data. L-JG performed the CMR imaging, LGE data collection, and interpretation. GS performed the statistical analysis. SS, JK, GS, and L-JG wrote sections of the paper. All authors contributed to manuscript revision and read and approved the submitted version.

## Funding

This work was part of the Ph.D. dissertation of SS, supported by a Zoetis-Morris Animal Foundation fellowship (grant number D14CA-903). The digital scanning of slides was supported through generous equipment sharing by the GI Lab at Texas A&M University. Amanda Bettis and Heather Heath-Barnett provided dedicated care of the dogs and maintenance of the colony.

## Conflict of Interest

JK reports personal fees from Solid Biosciences as a paid consultant, outside the submitted work. The remaining authors declare that the research was conducted in the absence of any commercial or financial relationships that could be construed as a potential conflict of interest.

## Publisher's Note

All claims expressed in this article are solely those of the authors and do not necessarily represent those of their affiliated organizations, or those of the publisher, the editors and the reviewers. Any product that may be evaluated in this article, or claim that may be made by its manufacturer, is not guaranteed or endorsed by the publisher.
