# Supplementary material for: Natural History of Histopathologic Changes in Cardiomyopathy of Golden Retriever Muscular Dystrophy
Source: Front Vet Sci. 2022 Feb 17;8:759585. doi: 10.3389/fvets.2021.759585 (PMC8892215; doi:10.3389/fvets.2021.759585)
Supplement: Supplementary file 1 [file Data_Sheet_1.docx]

Supplemental Data 1

**1.1 Background area Macro**

Input select file

Output select File or make new file

Select Output Format:TIFF

Add Macro Code:choose [Select from list]

run("Colour Deconvolution", "vectors=[Masson Trichrome]");

close();

close();

setAutoThreshold("Default");

//run("Threshold...");

setThreshold(0, 200);

run("Measure");

close();

selectWindow("Colour Deconvolution");

close();

**1.2 Fibrosis macro**

Input select file

Output select File or make new file

Select Output Format:TIFF or JPEG

Add Macro Code:choose [Select from list]

run("Colour Deconvolution", "vectors=[Masson Trichrome]");

close();

close();

setAutoThreshold("Default");

//run("Threshold...");

setThreshold(0, 120);

run("Measure");

close();

selectWindow("Colour Deconvolution");

close();

**Supplemental Figure 1.** Representative trichrome stained sections of heart show the semi-quantitative grade for approximate percentage of cross-sectional area affected by histopathologic lesions: 0 = none, 1 = 1 to 10%, 2 = 11-20%, 3 = 21 to 30%, and 4 > 30%.

| **Supplemental Table 1. Signalment, gross measurements, and HW/BW ratios for all dogs.** | | | | | | | | | | | |
| --- | --- | --- | --- | --- | --- | --- | --- | --- | --- | --- | --- |
| **Age (m) and sex** |  | **Fixed HW (g)** | **BW (kg)** | **LV (cm)** | **RV (cm)** | **Septum (cm)** | **HW/BW (g/kg)** | **LV/RV** | **LV/Sept** | **LV/HW (mm/g)** |  |
| **GRMD** |  |  |  |  |  |  |  |  |  |  |  |
| 3 F |  | 50 | 7 | 0.6 | 0.3 | 0.7 | 7.14 | 0.50 | 0.86 | 0.12 |  |
| 3 F |  | 42 | 5.7 | 1 | 0.3 | 0.8 | 7.37 | 0.30 | 1.25 | 0.24 |  |
| 3 F |  | 41 | 6.6 | 0.7 | 0.3 | 0.7 | 6.21 | 0.43 | 1.00 | 0.17 |  |
| 3 M |  | 49 | 8 | 0.9 | 0.3 | 1.1 | 6.13 | 0.33 | 0.82 | 0.18 |  |
| 3 M |  | 49 | 8.5 | 0.8 | 0.3 | 0.6 | 5.76 | 0.38 | 1.33 | 0.16 |  |
| 10 M |  | 116 | 27 | 1 | 0.6 | 1.1 | 4.30 | 0.60 | 0.91 | 0.09 |  |
| 11 F |  | 90 | 9.7 | 1.1 | 0.4 | 1.3 | 9.28 | 0.36 | 0.85 | 0.12 |  |
| 11 M |  | 130 | 15.6 | 1.5 | 0.7 | 1.5 | 8.33 | 0.47 | 1.00 | 0.12 |  |
| 12 F |  | 126 | 30 | 1.4 | 0.7 | 1.4 | 4.20 | 0.50 | 1.00 | 0.11 |  |
| 12 F |  | 118 | 15.1 | 1.3 | 0.6 | 1.1 | 7.81 | 0.46 | 1.18 | 0.11 |  |
| 12 F |  | 109 | 13.3 | 1.1 | 0.6 | 1 | 8.20 | 0.55 | 1.10 | 0.10 |  |
| 12 M |  | 80 | 13 | 0.9 | 0.4 | 0.8 | 6.15 | 0.44 | 1.13 | 0.11 |  |
| 15 M |  | 81 | 9.3 | 0.8 | 0.4 | 0.9 | 8.71 | 0.50 | 0.89 | 0.10 |  |
| 17 M |  | 98 | 12.5 | 1.2 | 0.4 | 1 | 7.84 | 0.33 | 1.20 | 0.12 |  |
| 21 F |  | 98 | 24.3 | 0.7 | 0.3 | 1 | 4.03 | 0.43 | 0.70 | 0.07 |  |
| 21 F |  | 102 | 21 | 1.1 | 0.45 | 1 | 4.86 | 0.41 | 1.10 | 0.11 |  |
| 28 F |  | 132 | 13.8 | 1.8 | 0.4 | 1.3 | 9.57 | 0.22 | 1.38 | 0.14 |  |
| 36 M |  | 132 | 7.9 | 1.1 | 0.4 | 0.9 | 16.71 | 0.36 | 1.22 | 0.08 |  |
| 38 M |  | 188 | 21.9 | 1.3 | 0.5 | 1.1 | 8.58 | 0.38 | 1.18 | 0.07 |  |
| 40 F |  | 160 | 5.1 | 1.3 | 0.5 | 1 | 31.37 | 0.60 | 1.00 | 0.08 |  |
| 40 F |  | 164 | 13.8 | 1 | 0.6 | 1 | 11.88 | 0.38 | 1.30 | 0.06 |  |
| 40 M |  | 158 | 27 | 1.2 | 0.4 | 0.9 | 5.85 | 0.33 | 1.33 | 0.08 |  |
| 45 F |  | 238 | 17.3 | 1 | 0.4 | 0.8 | 13.76 | 0.40 | 1.25 | 0.04 |  |
| 51 M |  | 132 | 17.6 | 1 | 0.8 | 0.4 | 7.50 | 0.80 | 2.50 | 0.08 |  |
| 76 M |  | 192 | 28.3 | 1.1 | 0.5 | 0.8 | 6.78 | 0.45 | 1.38 | 0.06 |  |
| 76 M |  | 194 | 21.9 | 1.5 | 0.5 | 1.3 | 8.86 | 0.33 | 1.15 | 0.08 |  |
| **Carrier** |  |  |  |  |  |  |  |  |  |  |  |
| 3 F |  | 62 | 14.9 | 1.2 | 0.6 | 1 | 4.16 | 0.50 | 1.20 | 0.19 |  |
| 12 F |  | 160 | 21.3 | 1.5 | 0.6 | 1.5 | 7.51 | 0.40 | 1.00 | 0.09 |  |
| 46 F |  | 202 | 15.1 | 1.6 | 0.7 | 1.3 | 13.38 | 0.44 | 1.23 | 0.08 |  |
| 56 F |  | 272 | 11.8 | 1.4 | 0.7 | 1.1 | 23.05 | 0.50 | 1.27 | 0.05 |  |
| 65 F |  | 182 | 13.5 | 1.2 | 0.7 | 1.2 | 13.48 | 0.58 | 1.00 | 0.07 |  |
| **Normal** |  |  |  |  |  |  |  |  |  |  |  |
| 22 F |  | 210 | 25.8 | 2 | 1 | 1.9 | 8.14 | 0.50 | 1.05 | 0.10 |  |
| 36 F |  | 186 | 23.8 | 1.7 | 0.8 | 1.7 | 7.82 | 0.47 | 1.00 | 0.09 |  |
| 46 F |  | 201 | 24.3 | 1.5 | 0.7 | 1.8 | 8.27 | 0.47 | 0.83 | 0.07 |  |
| 61 M |  | 236 | 26.9 | 2 | 1.1 | 2 | 8.77 | 0.53 | 0.95 | 0.08 |  |
| 75 F |  | 208 | 21 | 1.9 | 0.8 | 1.7 | 9.90 | 0.55 | 1.00 | 0.09 |  |
| 102 F |  | 224 | 28 | 1.9 | 1 | 2 | 8.00 | 0.42 | 1.12 | 0.08 |  |
| 124 M |  | -- | 25.8 | 2 | 0.9 | 2.3 | -- | 0.45 | 0.87 | -- |  |

| **Supplemental Table 2**. Semi-quantitative cross-sectional area score for LV and RV sections with means. | | | | | | | | | | | | | | | | | | | | | |
| --- | --- | --- | --- | --- | --- | --- | --- | --- | --- | --- | --- | --- | --- | --- | --- | --- | --- | --- | --- | --- | --- |
| **Age (m)** | **Basal anterior** | **Basal anteroseptal** | **basal inferoseptal** | **basal inferior** | **basal inferolateral** | **basal anterolateral** | **mid anterior** | **mid anteroseptal** | **mid inferoseptal** | **mid inferior** | **mid inferolateral** | **mid anterolateral** | **apical anterior** | **apical septal** | **apical inferior** | **apical lateral** | **Apex** | **RV basal anterior** | **RV basal inferior** | **RV mid anterior** | **RV mid inferior** |
| **GRMD** | | | | | | | | | | | | | | | | | | | | | |
| 3 | 0 | 0 | 0 | 0 |  | 0 | 0 | 0 | 0 | 0 | 1 | 1 | 1 | 0 | 0 | 0 | 1 | 0 | 0 | 0 | 0 |
| 3 | 0 | 0 | 0 | 0 | 0 |  | 0 | 0 | 0 | 1 | 0 | 0 | 1 | 1 | 0 | 0 | 1 | 1 | 1 | 1 | 1 |
| 3 | 1 | 1 | 1 | 1 | 0 | 0 | 0 | 0 | 0 | 1 | 0 |  | 0 | 1 | 1 | 0 | 1 | 1 | 1 |  | 1 |
| 3 | 0 | 0 | 0 | 1 | 1 |  | 0 | 0 | 0 | 0 |  | 0 | 0 | 0 | 0 | 0 | 1 | 0 | 0 | 0 | 0 |
| 3 | 0 | 0 | 0 | 0 | 0 | 0 | 1 | 0 | 0 | 0 | 0 |  | 1 | 0 | 0 |  |  | 1 | 1 | 0 | 0 |
| 10 | 0 | 0 | 0 | 0 | 0 | 0 | 0 | 1 | 0 | 0 | 0 | 1 | 0 | 0 | 0 | 0 | 0 | 0 | 0 | 1 | 1 |
| 11 | 1 | 1 | 1 | 3 |  | 1 | 2 | 1 | 1 | 1 | 1 | 1 | 2 | 1 | 0 | 0 | 1 | 1 | 1 | 1 | 0 |
| 10 | 1 | 0 | 1 | 0 | 0 |  | 0 | 0 | 0 | 0 | 0 | 0 | 0 | 1 | 0 | 1 | 1 | 0 | 1 | 0 | 0 |
| 12 | 1 | 2 | 1 | 1 | 1 | 3 | 1 | 2 | 1 | 1 | 1 | 2 | 2 | 2 | 1 | 2 | 1 | 1 | 2 |  |  |
| 12 | 1 | 1 | 0 | 1 | 1 | 1 | 1 | 1 | 0 | 1 | 1 | 0 | 2 | 1 | 0 | 0 | 0 | 1 | 1 | 1 | 1 |
| 12 | 1 |  | 1 | 2 | 1 | 4 | 1 | 1 | 1 | 1 | 1 | 2 | 1 | 1 | 0 | 0 | 0 |  | 4 |  | 3 |
| 12 | 0 | 1 | 1 | 0 | 0 | 1 | 1 | 0 | 1 | 1 | 0 | 1 | 1 | 2 | 1 | 1 | 1 | 1 | 1 | 1 | 1 |
| 15 | 0 | 1 | 1 | 1 | 1 | 1 | 0 | 0 | 0 | 1 | 0 | 1 | 0 | 0 | 1 | 1 | 1 | 1 | 1 | 1 | 1 |
| 17 | 0 | 0 | 0 | 0 | 0 | 0 | 0 | 0 | 0 | 0 | 0 | 0 | 0 | 0 | 0 | 0 | 0 | 0 | 0 | 0 | 0 |
| 21 | 1 | 2 | 1 | 1 | 1 | 1 | 1 | 2 | 1 | 1 | 3 | 1 | 2 | 1 | 1 | 3 | 1 | 1 | 1 | 1 | 0 |
| 21 | 0 | 1 | 1 | 1 | 1 | 0 | 1 | 2 | 2 | 1 |  | 1 | 1 | 1 | 1 | 0 | 1 | 1 | 1 | 1 | 1 |
| 28 | 1 | 1 | 2 | 2 | 1 | 2 |  |  |  | 4 |  | 3 | 1 | 0 | 1 | 2 | 2 | 4 | 3 |  |  |
| 36 | 1 | 1 | 1 | 1 | 1 | 1 | 1 | 1 | 1 | 1 | 1 | 1 | 1 | 1 | 1 | 1 | 0 | 1 | 1 | 1 | 1 |
| 38 | 1 | 1 | 2 | 1 | 2 | 2 | 1 | 1 | 1 | 1 | 2 | 1 | 1 | 1 | 1 | 1 | 1 | 1 | 1 | 1 | 1 |
| 40 | 1 | 2 | 1 | 1 | 1 | 3 | 2 | 1 | 1 | 2 | 2 |  |  | 1 | 1 | 2 | 1 | 3 | 3 | 2 | 3 |
| 40 | 2 | 2 | 1 | 1 | 3 |  | 2 | 1 | 1 | 1 | 3 | 2 | 2 | 2 | 2 | 2 | 2 | 4 | 2 | 2 | 2 |
| 40 | 1 | 1 | 1 | 1 | 2 | 3 | 3 | 1 | 1 | 1 | 2 | 1 | 3 | 1 | 1 | 3 | 2 | 4 | 2 | 1 | 4 |
| 45 | 1 | 1 | 2 | 3 | 2 | 3 | 3 | 2 | 1 | 2 | 2 |  | 3 | 2 |  | 3 | 3 | 3 | 4 | 1 | 2 |
| 51 | 2 | 3 | 4 | 1 | 2 | 2 | 2 | 2 | 3 | 3 | 2 | 2 | 3 | 3 | 1 | 3 | 2 | 2 | 4 | 2 | 2 |
| 76 |  |  |  |  |  |  | 1 | 1 | 1 | 1 | 1 | 1 | 1 | 1 | 2 | 1 | 1 | 1 | 1 | 1 | 1 |
| 76 | 1 | 1 | 1 | 1 | 0 | 1 | 1 | 1 | 1 | 1 | 1 | 1 | 2 | 1 | 1 | 1 | 1 | 1 | 1 | 1 | 1 |
| Mean | 0.7 | 1.0 | 1.0 | 1.0 | 0.9 | 1.4 | 1.0 | 0.8 | 0.7 | 1.0 | 1.0 | 1.0 | 1.2 | 1.0 | 0.7 | 1.1 | 1.0 | 1.4 | 1.5 | 0.9 | 1.1 |
| **Carrier** | | | | | | | | | | | | | | | | | | | | | |
| 3 |  |  |  |  |  |  | 0 | 0 | 0 | 0 |  |  | 0 |  | 0 |  |  |  |  | 0 | 1 |
| 12 | 0 | 0 | 0 | 0 | 0 | 1 | 0 | 0 | 0 | 0 | 0 |  | 0 | 0 | 0 |  | 0 | 0 | 0 | 0 | 0 |
| 46 | 2 | 1 | 1 | 1 | 1 |  | 1 | 1 | 2 | 1 | 2 | 2 | 2 | 1 | 2 | 1 | 1 | 1 | 1 | 1 | 3 |
| 56 |  |  | 0 | 0 | 0 | 0 | 0 | 0 | 0 | 0 | 1 | 1 | 0 | 0 | 1 | 0 | 0 | 1 | 1 | 1 | 1 |
| 65 | 1 | 1 | 1 | 1 | 1 | 0 | 1 | 1 | 0 | 1 |  | 0 | 1 | 1 | 1 | 1 | 1 | 1 | 1 | 0 | 1 |
| Mean | 1 | 0.7 | 0.5 | 0.5 | 0.5 | 0.3 | 0.4 | 0.4 | 0.4 | 0.4 | 1 | 1 | 0.6 | 0.5 | 0.8 | 0.7 | 0.5 | 0.75 | 0.75 | 0.4 | 1.2 |
| **Normal** | | | | | | | | | | | | | | | | | | | | | |
| 22 | 0 | 0 | 0 | 1 | 1 | 0 | 1 | 0 | 0 | 0 | 0 | 0 | 0 | 1 | 1 | 0 | 1 | 1 | 0 | 1 | 0 |
| 36 | 0 | 0 | 0 | 0 | 0 | 0 | 0 | 0 | 0 | 0 | 0 | 0 | 0 | 0 | 0 | 0 | 0 | 0 | 0 | 0 | 0 |
| 46 | 1 | 1 | 1 | 1 |  | 1 | 0 | 1 | 1 | 1 |  | 0 | 0 | 0 | 1 | 1 | 0 | 0 | 1 | 1 | 1 |
| 61 | 0 | 0 | 1 | 0 | 0 | 1 | 0 | 0 | 0 | 0 | 0 | 0 | 0 | 0 | 0 | 0 | 1 | 0 | 0 | 0 | 0 |
| 75 | 0 | 0 | 0 | 1 | 0 | 0 | 1 | 0 | 0 | 0 | 1 | 1 | 0 | 0 | 0 | 0 | 1 | 0 | 1 | 1 |  |
| 102 | 0 | 0 | 0 | 1 | 0 | 0 | 0 | 0 | 0 | 0 | 0 | 0 | 0 | 0 | 1 | 0 | 0 | 0 | 0 | 0 | 0 |
| 124 | 1 | 1 | 0 | 1 | 1 | 0 | 0 | 0 | 0 | 0 |  | 0 | 1 | 1 | 1 | 0 | 1 | 0 | 0 | 1 | 0 |
| Mean | 0.3 | 0.3 | 0.3 | 0.7 | 0.3 | 0.3 | 0.3 | 0.1 | 0.1 | 0.1 | 0.2 | 0.1 | 0.1 | 0.3 | 0.6 | 0.1 | 0.6 | 0.1 | 0.3 | 0.6 | 0.2 |
| Numbers are a semi-quantitative grade for approximate percent of cross-sectional area affected by histopathologic lesions, generally following the system described by Kane et al. 2013 [34]: 0 = none, 1 = 1 to 10%, 2 = 11-20%, 3 = 21 to 30%, and 4 > 30%. Color intensity from white (0) to red (4) visually highlights the different values. Gray squares lack a value. | | | | | | | | | | | | | | | | | | | | | |

| **Supplemental Table 3**. Trichrome fibrosis quantification for LV and RV sections with means. | | | | | | | | | | | | | | | | | | | | |
| --- | --- | --- | --- | --- | --- | --- | --- | --- | --- | --- | --- | --- | --- | --- | --- | --- | --- | --- | --- | --- |
| **Age (m)** | **Basal anterior** | **Basal anteroseptal** | **basal inferoseptal** | **basal inferior** | **basal inferolateral** | **basal anterolateral** | **mid anterior** | **mid anteroseptal** | **mid inferoseptal** | **mid inferior** | **mid inferolateral** | **mid anterolateral** | **apical anterior** | **apical septal** | **apical inferior** | **apical lateral** | **RV basal anterior** | **RV basal inferior** | **RV mid anterior** | **RV mid inferior** |
| **GRMD** | | | | | | | | | | | | | | | | | | | | |
| 3 | 5 | 3 | 10 | 3 |  | 2 |  | 2 | 20 | 3 | 3 | 3 | 4 | 4 | 2 | 4 | 7 |  | 2 | 20 |
| 3 | 7 | 7 | 3 | 5 | 7 |  | 6 | 8 | 14 | 5 | 4 | 3 | 6 | 5 | 3 | 11 | 5 | 27 | 8 | 14 |
| 3 | 8 | 7 | 6 | 10 | 8 | 6 | 7 | 5 | 7 | 8 | 9 |  | 6 | 3 | 16 | 7 | 7 | 6 |  | 7 |
| 3 | 13 | 4 | 4 | 10 | 12 |  | 6 | 3 | 5 | 8 |  | 3 | 6 | 6 | 7 | 8 | 8 | 9 | 7 | 7 |
| 3 | 15 | 8 | 10 | 8 | 14 | 13 | 13 | 9 | 7 | 10 | 12 |  | 11 | 10 | 19 |  | 15 | 14 | 9 | 7 |
| 10 | 6 | 4 | 2 | 5 | 4 | 4 | 3 | 4 | 3 | 4 | 3 | 4 | 3 | 3 | 5 | 5 | 6 | 7 | 6 | 10 |
| 11 | 4 | 4 | 2 | 6 |  | 4 | 7 | 8 | 2 | 2 | 2 | 2 | 5 | 6 | 3 | 2 | 4 | 6 | 8 | 2 |
| 10 | 7 | 4 | 2 | 3 | 4 |  | 9 | 4 | 3 | 4 | 1 | 3 | 3 | 3 | 2 | 3 | 5 | 4 | 4 | 3 |
| 12 | 5 | 5 | 3 | 5 | 5 | 13 | 5 | 7 | 3 | 5 | 4 | 3 | 3 | 7 | 4 | 8 | 4 | 5 |  |  |
| 12 | 5 | 4 | 2 | 9 | 5 | 8 | 5 | 5 | 4 | 14 | 7 | 6 | 4 | 3 | 4 | 14 | 17 | 12 | 7 | 8 |
| 12 | 1 |  | 3 | 3 | 3 | 3 | 3 | 2 | 3 | 2 | 3 | 2 | 3 | 3 | 3 | 1 |  | 2 |  | 5 |
| 12 | 5 | 3 | 4 | 4 | 3 | 3 | 7 | 5 | 4 | 4 | 4 | 3 | 5 | 16 | 4 | 4 | 6 | 4 | 7 | 6 |
| 15 | 9 | 7 | 5 | 16 | 12 | 6 | 6 | 5 | 7 | 10 | 7 | 7 | 7 | 14 | 13 | 10 | 14 | 14 | 17 | 17 |
| 17 | 6 | 6 | 6 | 7 | 9 | 5 | 7 | 6 | 3 | 5 | 5 | 11 | 4 | 6 | 5 | 5 | 8 | 6 | 8 | 11 |
| 21 | 10 | 10 | 10 | 7 | 7 | 7 | 3 | 15 | 3 | 2 | 3 | 2 | 3 | 3 | 2 | 2 | 18 | 18 | 15 | 3 |
| 21 | 5 | 14 | 7 | 7 | 5 | 5 | 5 | 7 | 3 | 35 |  | 8 | 5 | 8 | 5 | 4 | 13 | 13 | 6 | 5 |
| 28 | 3 | 3 | 5 | 5 | 6 | 4 |  |  |  | 11 |  | 17 | 2 | 1 | 1 | 1 | 10 | 11 |  |  |
| 36 | 8 | 11 | 8 | 6 | 5 | 9 | 8 | 5 | 5 | 7 | 9 | 5 | 9 | 9 | 7 | 6 | 9 | 12 | 7 | 9 |
| 38 | 6 | 7 | 7 | 8 | 9 | 12 | 6 | 3 | 6 | 5 | 10 | 6 | 7 | 7 | 3 | 8 | 7 | 8 | 4 | 4 |
| 40 | 6 | 9 | 8 | 7 | 7 | 5 |  | 5 | 4 | 5 | 5 |  |  | 8 | 3 | 5 | 5 | 5 | 6 | 10 |
| 40 | 10 | 7 | 6 | 30 | 7 |  | 9 | 7 | 5 | 6 | 11 |  | 7 | 8 | 6 | 5 | 9 | 7 | 6 | 10 |
| 40 | 9 | 11 | 4 | 10 | 9 | 9 | 10 | 12 | 6 | 6 | 8 | 6 | 12 | 5 | 7 | 7 | 10 | 10 | 28 | 41 |
| 45 |  | 5 | 11 | 11 | 8 | 15 | 15 | 7 | 5 | 9 | 13 |  | 19 | 8 |  | 13 | 11 | 8 | 4 | 5 |
| 51 | 8 | 7 | 11 | 7 | 8 | 11 | 8 | 10 | 11 | 6 | 6 | 9 | 12 | 11 | 7 | 10 | 9 | 12 | 12 | 9 |
| 76 |  |  |  |  |  |  | 9 | 6 | 5 | 6 | 7 | 4 | 6 | 6 | 11 | 7 | 7 | 7 | 6 | 4 |
| 76 | 2 | 5 | 4 | 4 | 3 | 2 | 5 | 11 | 14 | 3 | 2 | 2 | 12 | 2 | 2 | 5 | 4 | 4 | 6 | 16 |

| Mean | | 6.8 | | 6.4 | | 5.7 | | 7.7 | | 6.9 | | 7.0 | | 6.9 | | 6.4 | | 6.1 | | 7.1 | | 5.9 | 5.2 | 6.6 | 6.3 | 5.7 | 6.3 | 8.7 | 9.2 | 8.3 | 9.7 |
| --- | --- | --- | --- | --- | --- | --- | --- | --- | --- | --- | --- | --- | --- | --- | --- | --- | --- | --- | --- | --- | --- | --- | --- | --- | --- | --- | --- | --- | --- | --- | --- |
| **Carrier** | | | | | | | | | | | | | | | | | | | | | | | | | | | | | | | |
| 3 | |  | |  | |  | |  | |  | |  | | 4 | | 3 | | 4 | | 4 | |  |  | 19 |  | 16 |  |  |  | 3 | 4 |
| 12 | | 5 | | 4 | | 3 | | 5 | | 4 | | 4 | | 5 | | 1 | | 2 | | 11 | | 2 |  | 2 | 5 | 4 |  | 5 |  | 4 | 3 |
| 46 | | 8 | | 5 | | 10 | | 6 | | 4 | |  | | 4 | | 8 | | 8 | | 8 | | 9 | 7 | 8 | 8 | 4 | 8 | 6 | 5 | 15 | 6 |
| 56 | |  | |  | | 7 | | 29 | | 5 | | 36 | | 5 | | 7 | | 3 | | 3 | | 3 | 3 | 3 | 8 | 7 | 3 | 7 | 8 | 5 | 4 |
| 65 | | 44 | | 12 | | 9 | | 12 | | 12 | | 13 | | 7 | | 7 | | 6 | | 4 | |  | 3 | 6 | 5 | 12 | 12 | 6 | 6 | 7 | 6 |
| Mean | 18 | | 7.4 | | 7.4 | | 13 | | 6.2 | | 17 | | 4.9 | | 5.2 | | 4.5 | | 6.0 | | 4.8 | | 4.7 | 7.7 | 6.4 | 8.8 | 7.8 | 6.2 | 6.6 | 6.6 | 4.7 |
| **Normal** | | | | | | | | | | | | | | | | | | | | | | | | | | | | | | | |
| 22 | | 6 | | 12 | | 3 | | 5 | | 6 | | 4 | | 10 | | 7 | | 15 | | 10 | | 14 | 12 | 6 | 4 | 4 | 4 | 5 | 4 | 9 | 8 |
| 36 | | 3 | | 5 | | 2 | | 3 | | 3 | | 2 | | 3 | | 2 | | 2 | | 3 | | 2 | 3 | 3 | 4 | 3 | 3 | 3 | 3 | 5 | 3 |
| 46 | | 4 | | 14 | | 6 | | 3 | |  | | 9 | | 4 | | 5 | | 5 | | 4 | |  | 5 | 5 | 7 | 4 | 6 | 5 | 7 | 7 | 5 |
| 61 | | 1 | | 1 | | 2 | | 1 | | 1 | | 1 | | 1 | | 1 | | 1 | | 5 | | 2 | 1 | 2 | 1 | 1 | 5 | 1 | 2 | 1 | 1 |
| 75 | | 2 | | 2 | | 2 | | 2 | | 2 | | 2 | | 3 | | 9 | | 2 | | 2 | | 1 | 4 | 2 | 2 | 2 | 2 | 2 | 2 | 2 | / |
| 102 | | 3 | | 1 | | 1 | | 3 | | 1 | | 2 | | 10 | | 2 | | 1 | | 2 | | 1 | 2 | 2 | 1 | 2 | 2 | 3 | 3 | 3 | 3 |
| 124 | | 3 | | 8 | | 8 | | 3 | | 4 | | 2 | | 3 | | 4 | | 13 | | 7 | |  | 15 | 3 | 4 | 2 | 3 | 3 | 2 | 6 | 2 |
| Mean | | 3.1 | | 6.2 | | 3.5 | | 2.9 | | 2.7 | | 3.2 | | 4.8 | | 4.2 | | 5.6 | | 4.6 | | 4.1 | 5.8 | 3.3 | 3.4 | 2.5 | 3.4 | 3.0 | 3.1 | 4.7 | 3.8 |
| Numbers are based on an assessment of positive (blue) staining for collagen in sections measured by assessment of jpeg images of each slide in ImageJ. Numbers are rounded to the nearest whole percentage. | | | | | | | | | | | | | | | | | | | | | | | | | | | | | | | |
