# Supplementary figures and images for: Natural History of Histopathologic Changes in Cardiomyopathy of Golden Retriever Muscular Dystrophy
Source: Front Vet Sci. 2022 Feb 17;8:759585. doi: 10.3389/fvets.2021.759585 (PMC8892215; doi:10.3389/fvets.2021.759585)

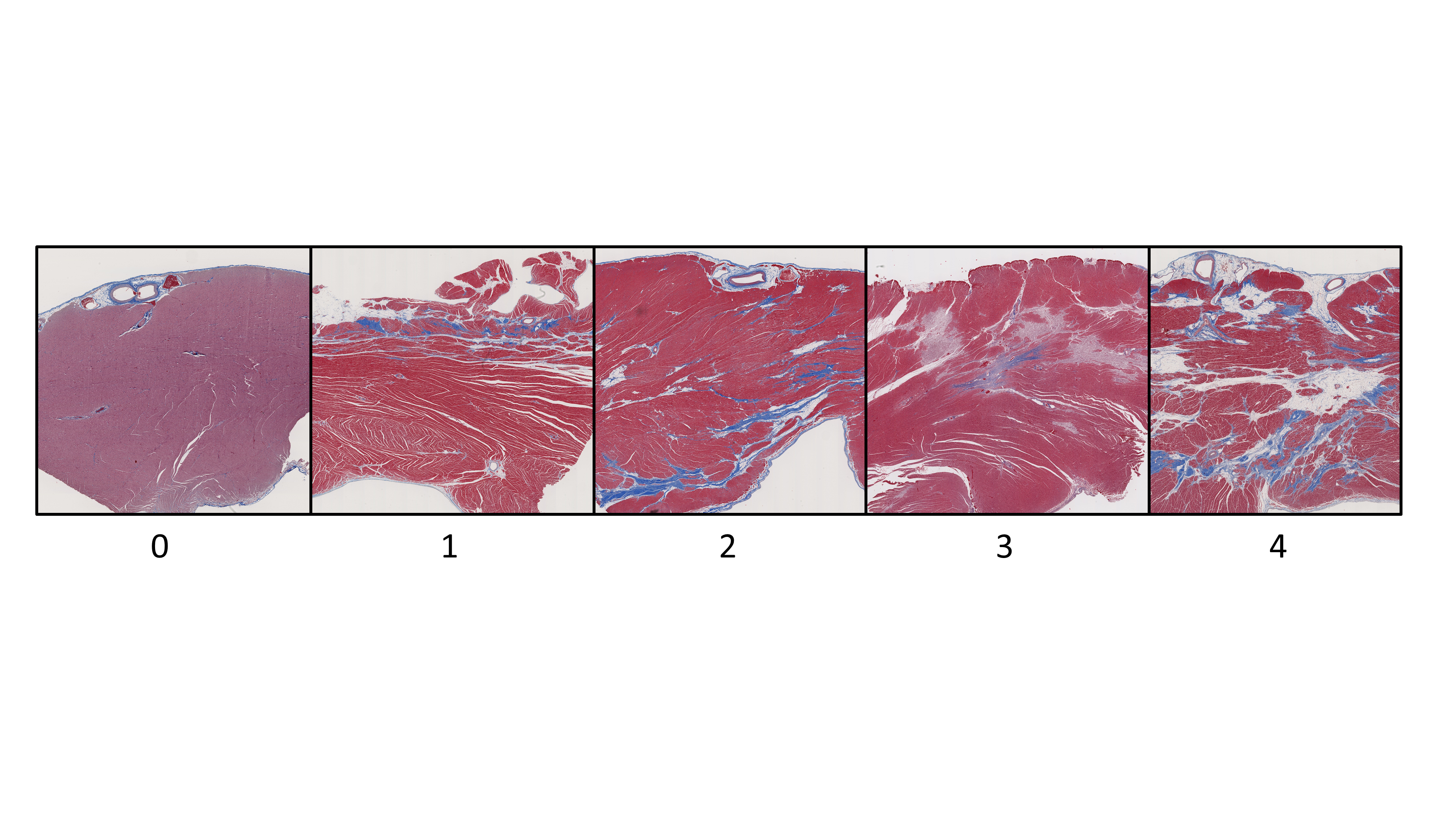

Supplement: Supplementary file 2 [file Image_1.TIF]
